# Supplementary material for: Learning curve and surgical outcome of robotic assisted colorectal surgery with ERAS program
Source: Sci Rep. 2022 Nov 29;12:20566. doi: 10.1038/s41598-022-24665-w (PMC9709162; doi:10.1038/s41598-022-24665-w)
Supplement: Supplementary file 1 — Supplementary Information 1. [file 41598_2022_24665_MOESM1_ESM.zip › LSC Raw data-Ñ[▒K/134/N_ñw╝╨░O▒Kñσ.pdf]

W83 / W83

臺中榮民總醫院

手術室護理記錄

[redacted]

基本資料

- 室別： 二醫手術室-31室，手術科別： CRS，手術類別： 常規，日期： 2021/08/26
- 進入時間： 1210 ； 麻醉開始時間： 1315 ； Time Out時間： 1310 ； 劃刀時間： 1312 ； 手術結束時間： 1540 送出時間： 1555 ；
- 麻醉方式： GE

  診斷：HEPATCI FLEXURE CA PROVED

  手術名稱：1.Laparoscopic right colectomy and anastomosis2.Biliary cholecystectomy3.4.

  手術局部用藥：NIil

  術前預防性抗生素：
  - 抗生素名稱一： Veterin 1GM IVD/由麻醉護理給予
  - 時間： 1305
  - 上次簽章: 林采儀

  ，追加預防性抗生素：
- 手術醫師： 蔣鋒帆
- 麻醉醫師： VS 賴慧卿
-
  - 刷手護士A ： 黃鈺琇，時間： 1210
  - 流動護士A ： 林采儀，時間： 1210，接替流動一： 彭馨慧，時間： 1500

皮膚狀況

A-V Shunt： 無

術前： 完整

記錄者： 上次簽章: 林采儀

術後： 完整

記錄者： 彭馨慧

手術準備

手術臥式 單一手術臥式

-
  - 手術臥式： 仰臥
  - 使用附件： 溫毯 38 ℃
  - 皮膚準備： 10%Aq Betadine，Hibiscrub，2% Chlorhexidine
  - 電燒： 使用電燒: 電極片位置： 其它： 床上迴路電極墊，記錄者： 上次簽章: 林采儀

止血帶T： 無

植入物

- 植入物名稱/號碼/數量/部位：Ethicon NTLC75/貳釘/colon
  Surgiwrap 130*200mm/pelvic/壹

，記錄者：上次簽章: 彭馨慧

標本送檢

- 細菌培養 無
- 細胞學 無
- 冰凍切片 無
- 病理標本

  組織名稱一：A-colon+gall bladder
- 截肢 無

標本交付 標本： 壹 袋，標本： 零 盒，標本： 零 瓶，細胞學： 零 管，Culture Tube： 零 管，被交付醫師： Fellow 楊曙榮，

- 記錄者： 彭馨慧
- 時間： 1550

傷口術後敷料

傷口敷料： 傷口敷料： 無

- 請於圖上標示部位：

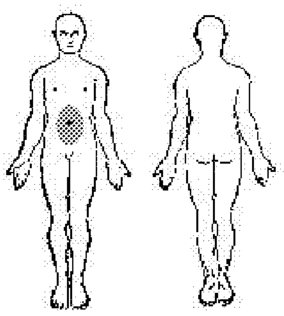

腹部直切及叁切口

- 傷口敷料： 白紗及Menpor覆蓋

導管

導管	天數	導管資訊	插入日期	插入時間	更換日期	拔除日期	拔除時間	拔除原因	預計拔除/更換	預計拔除/更換日期
A-line (1)	1	號碼:22 號; 部位:左手;	2021/08/26	1230						
Foley(1)	1	號碼:16 Fr.; 部位:膀胱;	2021/08/26	1235						
Sump NG Tube(1)	1	號碼:16 Fr.; 深度:53; 部位:胃;	2021/08/26	1240						

用物計數

- 手術中出血量： 少量 c.c.
- 手術中尿量： 300 c.c.
-
    - 其它： 無

用物計數： 多切口同時縫合

- 1.器械： 正確，2.縫針： 正確，3.刀片： 正確，4.紗布： 正確，5.腹部墊： 正確，6.紗球： 未使用，7.PEANUTS ： 未使用，8.紗條： 未使用，9.絲帶： 未使用，10.針頭： 未使用，11.Vessel loop： 未使用，12.Boots： 未使用，13.Jelco： 未使用，14.棉條： 未使用，
    - 第一次：
        - 刷手護士 ： 黃鈺琇
        - 流動護士： 上次簽章: 彭馨慧
        - 時間： 1500

  ,
    - 第二次：
        - 刷手護士 ： 黃鈺琇
        - 流動護士： 上次簽章: 彭馨慧
        - 時間： 1515

  ,
    - 第三次：
        - 刷手護士 ： 黃鈺琇
        - 流動護士： 上次簽章: 彭馨慧
        - 時間： 1525

備註

備註： NIL

- 記錄者： 上次簽章: 彭馨慧
- 時間： 1520

填寫護理人員 彭馨慧

正式摘要

W83 / W83

doctorRank:B
